# Supplementary material for: Trodusquemine enhances Aβ42 aggregation but suppresses its toxicity by displacing oligomers from cell membranes
Source: Nat Commun. 2019 Jan 15;10:225. doi: 10.1038/s41467-018-07699-5 (PMC6333784; doi:10.1038/s41467-018-07699-5)
Supplement: Supplementary file 1 — Supplementary Information [file 41467_2018_7699_MOESM1_ESM.pdf]

SUPPLEMENTARY INFORMATION

Trodusquemine enhances Aβ_{42} aggregation but suppresses its toxicity by displacing oligomers from cell membranes

Limbocker et al.

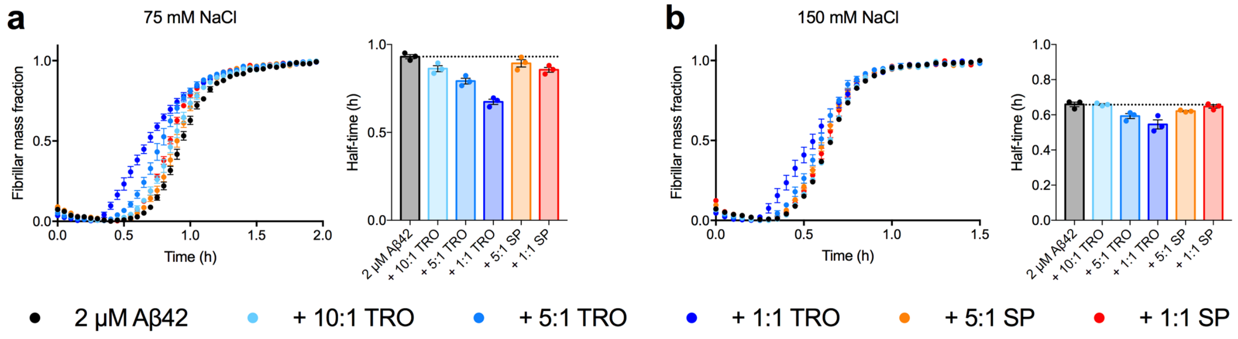

Supplementary Figure 1. The accelerative effects of trodusquemine and spermine at varying ionic strength. Normalized kinetic profiles of the aggregation and corresponding half-times of aggregation for 2 μM Aβ42 (5 mM sodium phosphate, 200 μM EDTA, pH 8) in the absence (black) and presence of trodusquemine (TRO) at ratios of Aβ42-to-trodusquemine of 10:1, 5:1 and 1:1 (light to dark blue) or spermine (SP) at ratios of Aβ42-to-spermine of 5:1 (orange) and 1:1 (red) in the presence of 75 mM NaCl (**a**) or 150 mM NaCl (**b**). All data represent mean ± standard error of the mean (s.e.m.) of three replicates.

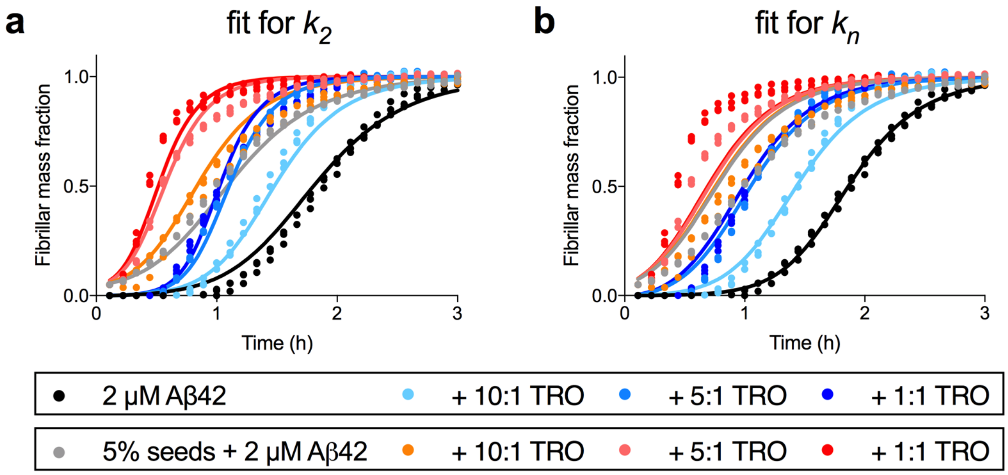

Supplementary Figure 2. Kinetic fitting of the Aβ_{42} aggregation traces in the presence of trodusquemine indicates an increase in k_2 but not k_n. Kinetic profiles of the aggregation of 2 μM Aβ_{42} in the absence (black) or presence of trodusquemine at ratios of Aβ_{42}-to-trodusquemine of 10:1, 5:1, 1:1, and 1:3 (light to dark blue) and kinetic profiles of the aggregation of 2 μM Aβ_{42} in the presence of 5% seed fibrils and in the absence (grey) or presence of trodusquemine at ratios of Aβ_{42}-to-trodusquemine of 10:1, 5:1, and 1:1 (orange to red). The unseeded (see Fig. 1b) and 5% seeded (see Fig. 1c) data were fit globally for an effect on (**a**) secondary nucleation (k_2) and (**b**) primary nucleation (k_n).

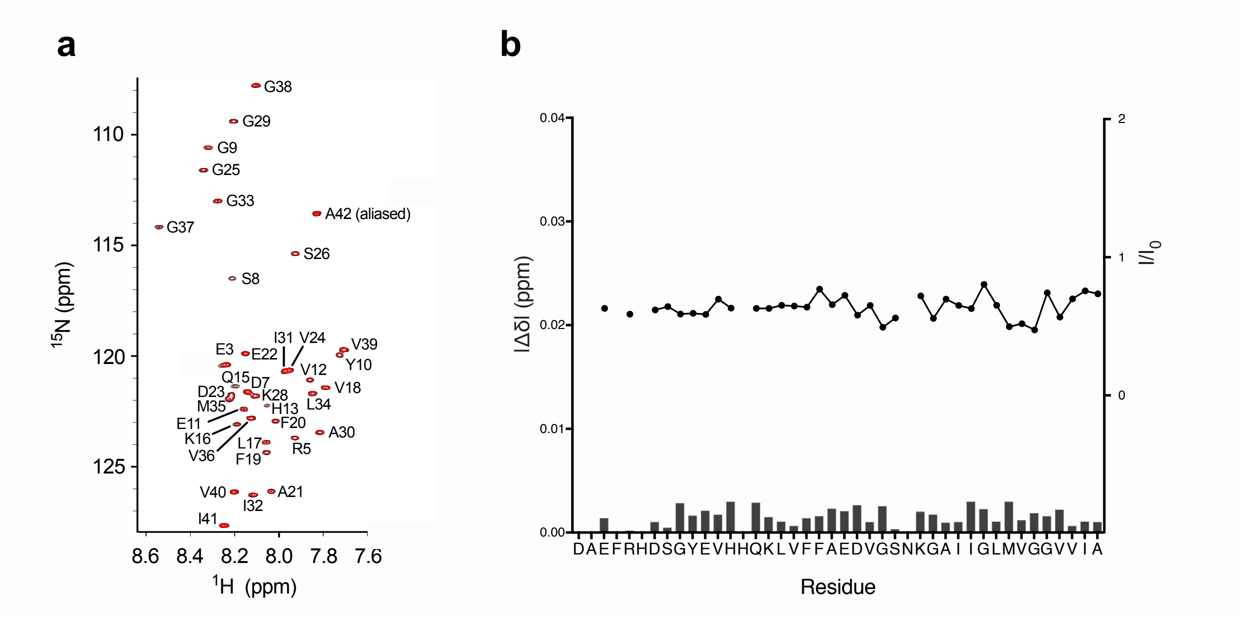

Supplementary Figure 3. Trodusquemine does not cause significant changes to the average conformation of monomeric Aβ_{42}. (**a**) ^{1}H-^{15}N-HSQC spectra of 50 µM ^{15}N-labeled Aβ_{42} in the absence (black) or presence (red) of 50 µM trodusquemine. 8 scans were taken for each spectra at 5 ˚C on a 500 MHz NMR. Residue assignments were taken from previous work[1]. (**b**) Changes in chemical shift values, |Δδ|, (bars, left axis) and peak intensities, I, (dots, right axis) of Aβ_{42} in the presence of trodusquemine relative to the condition in the absence of the aminosterol. All samples were prepared on ice in 5 mM sodium phosphate, pH 7.4. Please refer to Supplementary Table 1 for raw NMR data and the definition of |Δδ|.

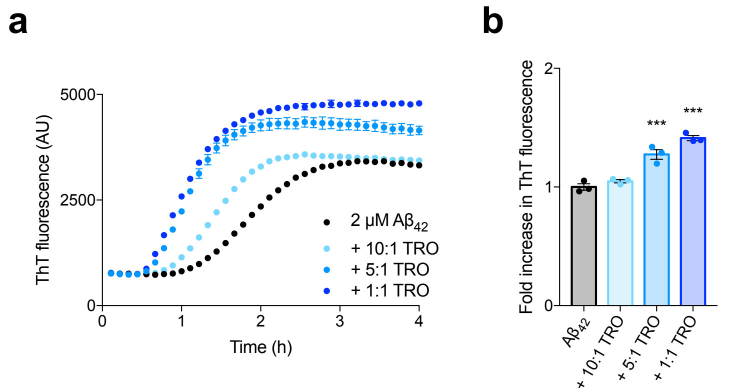

Supplementary Figure 4. The presence of trodusquemine moderately increases the plateau ThT fluorescence intensity at the end-point of aggregation. (**a**) Kinetic profiles of the aggregation of 2 μM Aβ_{42} in the absence (black) or presence of trodusquemine at ratios of Aβ_{42}-to-trodusquemine of 10:1, 5:1, 1:1 (light to dark blue). Normalized data are shown in Fig. 1b. (**b**) The fold increase in plateau ThT fluorescence signal after monomer depletion with increasing concentrations of trodusquemine. ***P<0.001 by one-way ANOVA followed by Bonferroni's multiple comparison test relative to the condition in the absence of trodusquemine. All data represent mean ± s.e.m. of three replicates.

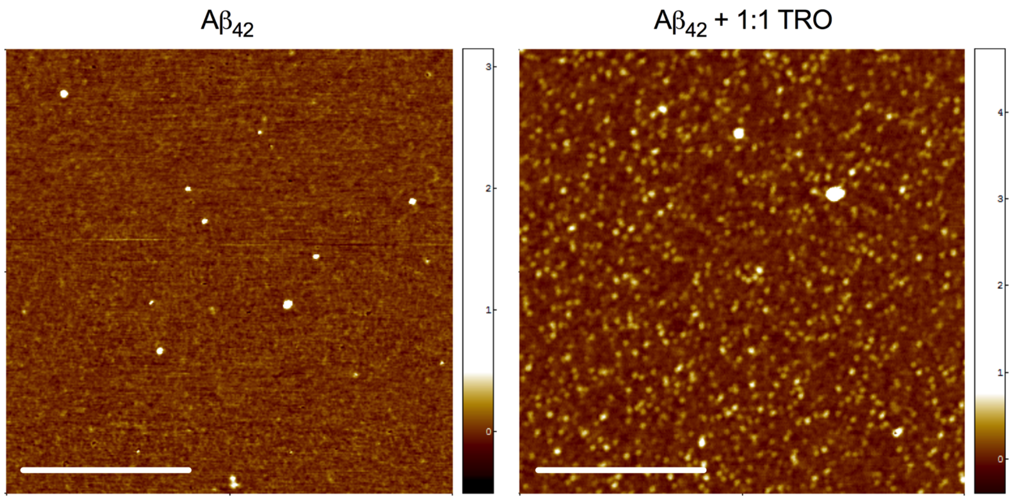

Supplementary Figure 5. Representative AFM images at t=0h of aggregation. $A\beta_{42}$ was deposited at a concentration of 2 μM in the absence (left) and presence of an equimolar concentration of trodusquemine (right). Mature aggregates were not observed at the initiation stage of aggregation, in agreement with the chemical kinetics data. Scale bars, 500 nm.

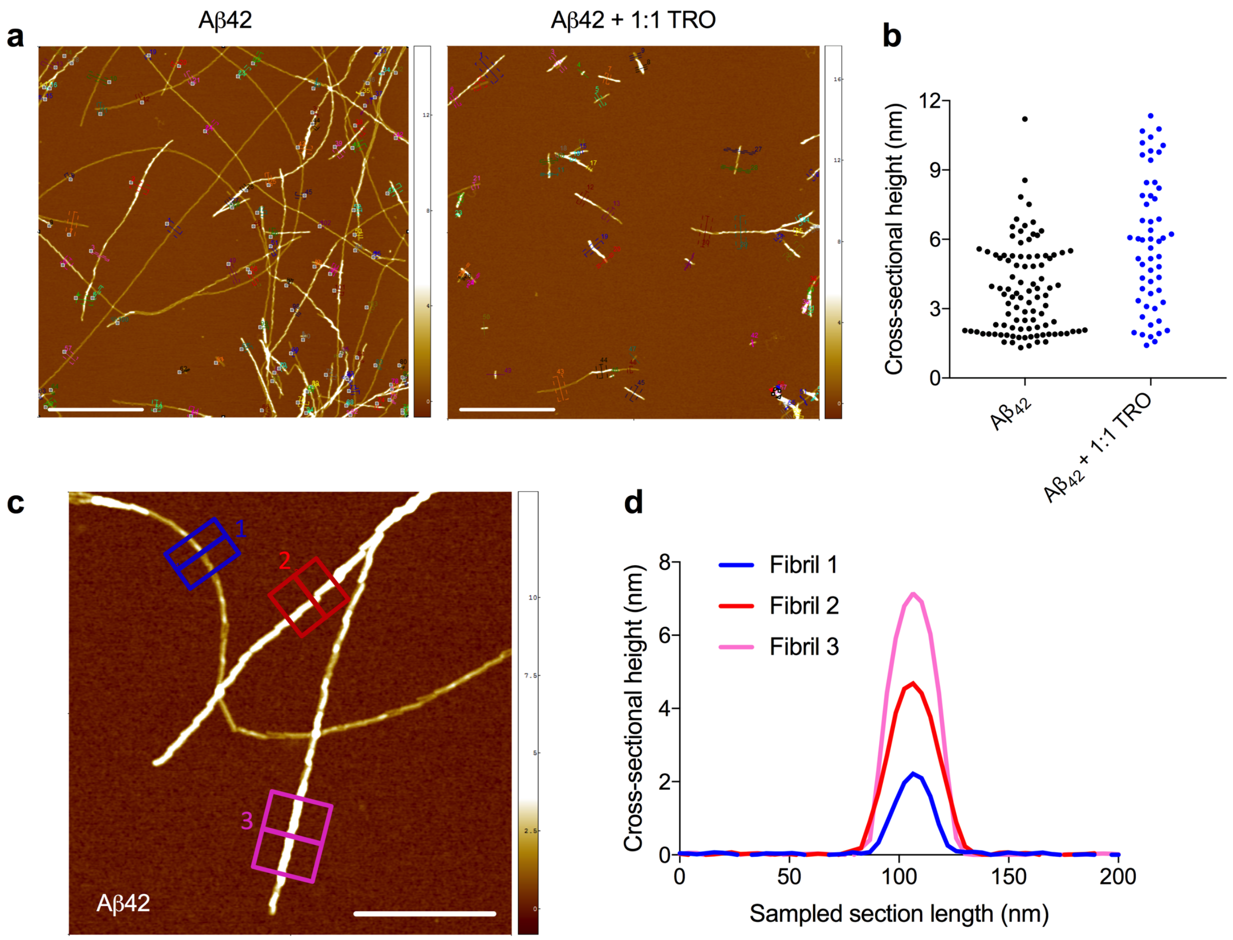

Supplementary Figure 6. Method of AFM height quantification. (**a**) Fibrils were imaged at constant high resolution and under consistent tip-sample interaction[2]. Samples were prepared after fibril formation from 2 µM monomeric Aβ42 with solution deposition on an APTES functionalized surface and analyzed using SPIP for Fig. 2. Scale bars, 1 µm. (**b**) Distributions of cross-sectional heights corresponding to the images in (**a**). (**c**) The method for determining fibril height is illustrated explicitly, with representative fibrils of various heights shown (different colors), in this case having formed in the absence of trodusquemine (**d**). Scale bar, 500 nm.

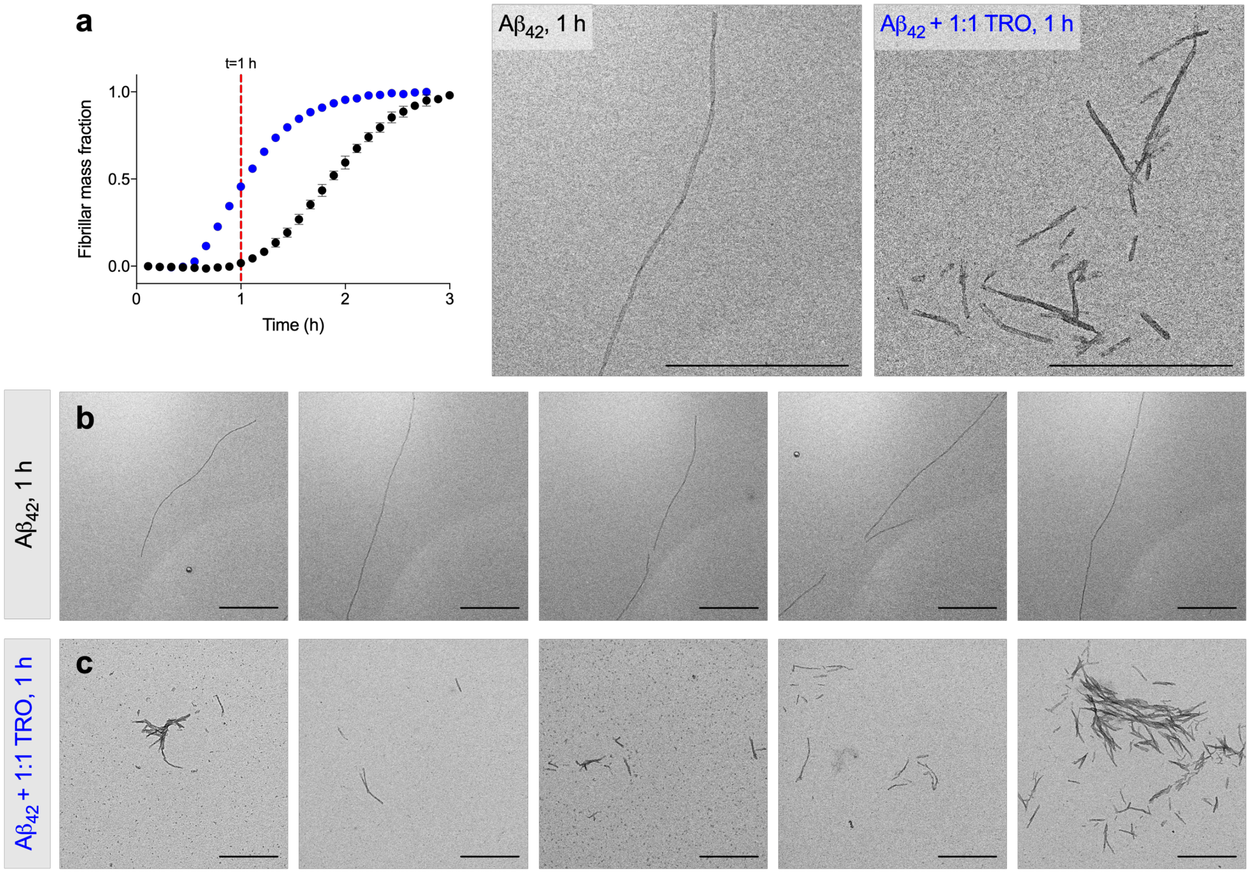

Supplementary Figure 7. Aβ42 fibril lengths during the aggregation reaction in the presence of trodusquemine are consistent with an increase in the rate of secondary nucleation. (a) Kinetic profiles of the aggregation of 2 μM Aβ42 in the absence (black) and presence of trodusquemine at a ratio of Aβ42-to-trodusquemine of 1:1 (blue). Samples were deposited on carbon-coated TEM grids and stained with uranyl acetate after incubation for 1 h, as indicated by a red bar in the ThT aggregation traces. This time point was selected to assess the morphology of the aggregates that form during the growth phase of the aggregation reaction. Mean ± s.e.m. of three replicates. Additional representative TEM images in the absence (**b**) and presence (**c**) of trodusquemine. Scale bars, 500 nm.

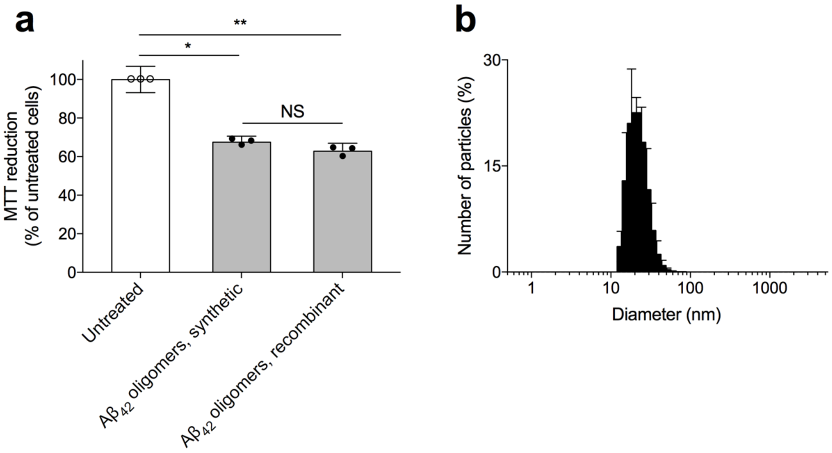

Supplementary Figure 8. Characterization of the cellular toxicity and heterogeneity of the Aβ$_{42}$ oligomer preparation. (**a**) Oligomers were produced as previously described[3] from synthetic or recombinant Aβ$_{42}$ (12 μM monomer equivalents) and added to the cell culture medium of SH-SY5Y cells for 24 h. Viability was determined by the ability of cells to reduce MTT. *P<0.05, **P<0.01, NS: not significant by Student's t-test. Data represent mean ± s.e.m. of three independent experiments. (**b**) Dynamic light scattering measurements of the oligomers at a concentration of 5 μM (monomer equivalents). Error bars indicate s.e.m. of three replicates.

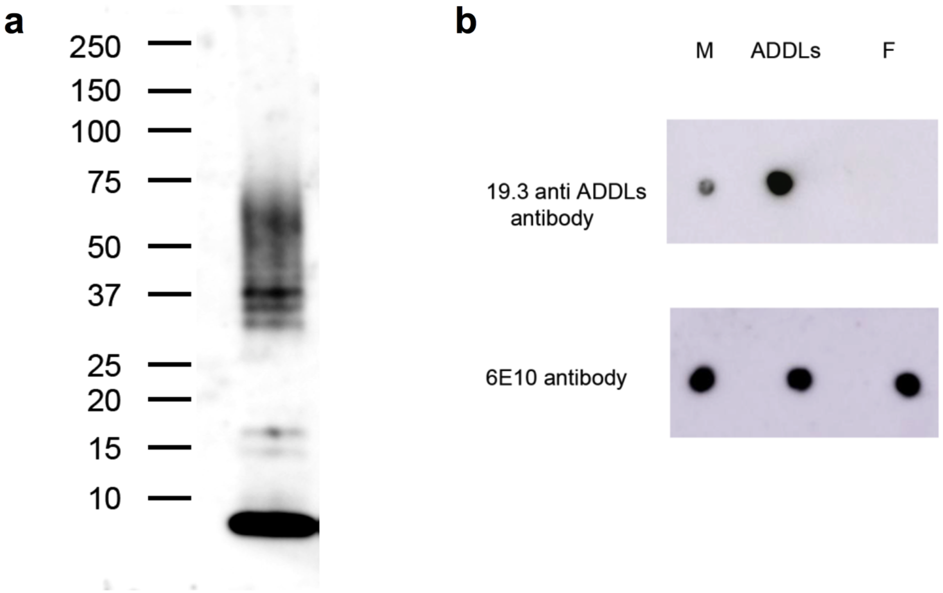

Supplementary Figure 9. Characterization of the Aβ_{42} oligomers. (**a**) ADDLs (2.25 µg) were produced as previously described[3] and separated by western blotting. (**b**) Dot-blot analysis of Aβ_{42} monomers (M), oligomers (ADDLs) and fibrils (F) using 2 µl/spot corresponding to 1 µg in monomer equivalents. Samples were probed with the ADDL-specific human anti-ADDLs therapeutic antibody (19.3) and the sequence specific 6E10 antibody.

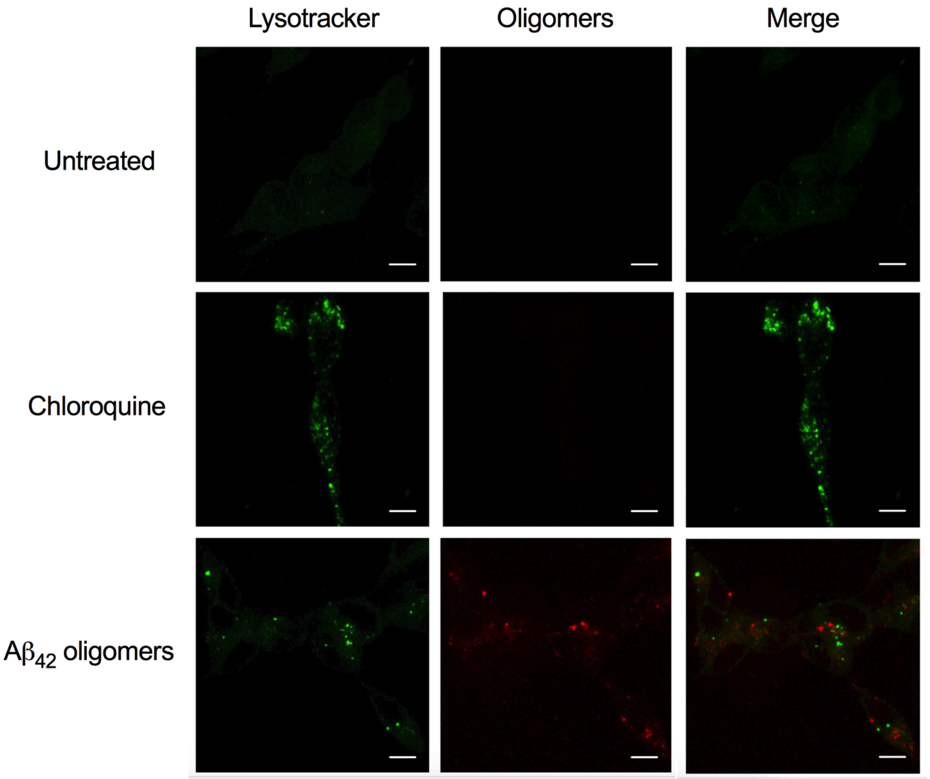

Supplementary Figure 10. Aβ_{42} oligomers do not significantly induce lysosome activity and do not colocalize with lysosomes. Oligomers were produced as previously described[3] from 647-labeled monomeric Aβ_{42} and incubated with cells for 15 min at a concentration of 1 μM. Cells were also treated with 50 μM chloroquine for 4 h as a positive control. Cells were washed and treated with 50 nM of LysoTracker Green DND-26 for 30 min. Red and green channels indicate oligomers and LysoTracker, respectively. Scale bars, 10 μm.

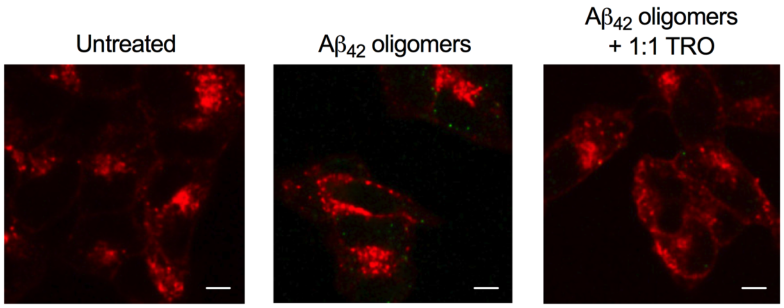

Supplementary Figure 11. Lack of Aβ_{42} oligomers internalized into the cytosol. The median planes of the cells corresponding to Fig. 3c were analyzed to monitor the intracellular fluorescence. No green fluorescence was observed. Given that the cells were not permeabilized and the antibody cannot cross the plasma membrane, the observed fluorescence observed in Fig. 3c is therefore a result of oligomers bound to the cell membranes. Scale bars, 10 μm.

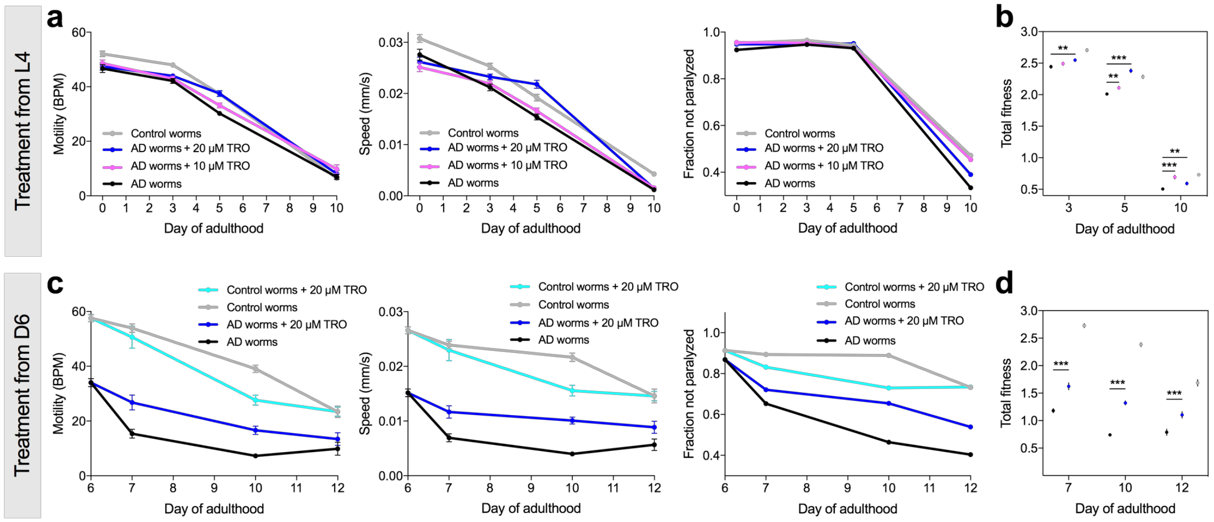

Supplementary Figure 12. Trodusquemine increases AD worm health. (**a**) Worms were incubated from the L4 stage of development in the absence (black) and presence of 10 µM (purple) and 20 µM (blue) doses of trodusquemine, and monitored from day 0 of adulthood (D0) to D10 for motility (left), speed of swimming (middle) and fraction not paralyzed (right). Untreated control worms (grey) are shown for comparison. N=262, 375, 342 and 170 for AD worms, 303, 311, 352 and 82 for AD worms+10 µM TRO, 245, 432, 309 and 107 for AD worms+20 µM TRO and 420, 389, 320 and 278 for Control worms at D0, D3, D5 and D10, respectively. (**b**) Total fitness scores over time corresponding to the sum of the readouts in (**a**). ***P*<0.01, ****P*<0.001 by Student's *t*-test. (**c**) Worms were also incubated from D6 in the absence or presence of a 20 µM dose of trodusquemine and monitored from D7 to D12. Treated control worms (cyan) are shown for comparison. N=105, 103 and 46 for AD worms, 88, 203 and 80 for AD worms+20 µM TRO, 84, 245 and 215 for Control worms+20 µM TRO and 434, 410 and 144 for Control worms at D7, D10 and D12, respectively. (**d**) Total fitness scores over time corresponding to the sum of the readouts in (**c**). ****P*<0.001 by Student's *t*-test. All data represent mean ± s.e.m. with the N values listed above. D5 in the early treatment experiment and D7 in late treatment experiment are shown as radar plots in Fig. 5, which are representative of three biological replicates that yielded similar results.

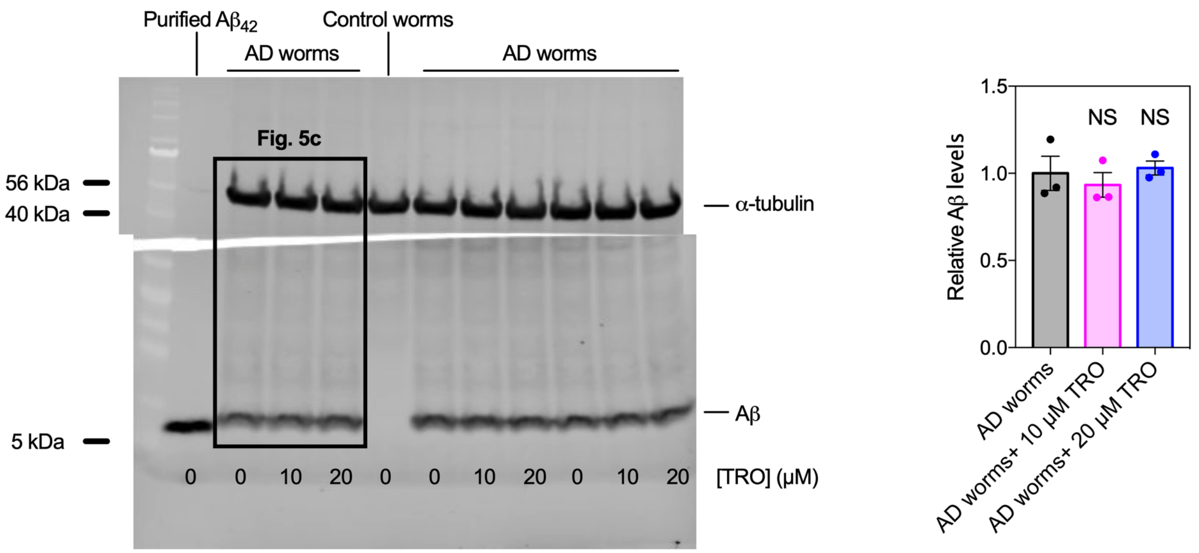

Supplementary Figure 13. Trodusquemine does not alter the total accumulation of Aβ_{42} in AD worms. Worms were incubated in the absence (black) and presence of 10 µM (purple) and 20 µM (blue) doses of trodusquemine and extracted at D5. Western blot measurements of the total accumulation of Aβ_{42} in the worm lysate. Control worms not expressing Aβ_{42} and purified monomeric Aβ_{42} were run as negative and positive controls, respectively. The quantification of Aβ_{42} levels after standardization relative to the signal of α-tubulin (ImageJ, gel analysis) is reproduced in Fig. 5d. Bars indicate mean ± s.e.m. of the three replicates shown above. NS: not significant by one-way ANOVA followed by Bonferroni's multiple comparison test relative to AD worms. Representative data from duplicate experiments are shown. The blot is unedited apart from aligning the top and bottom sections.

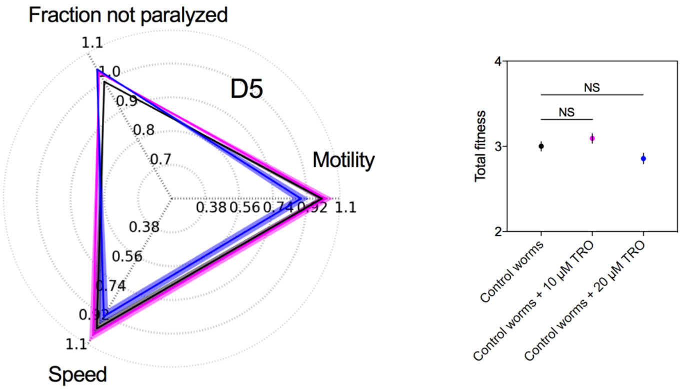

Supplementary Figure 14. Trodusquemine does not improve the fitness of wild-type *C. elegans*. The administration of trodusquemine at the L4 stage of development at concentrations sufficient to improve the fitness of $A\beta_{42}$-expressing worms (Fig. 5b) showed no benefit to the body bend frequency and speed of swimming of wild-type control worms, but did increase to a small extent the fraction of worms not paralyzed at D5. N=265, 234 and 193 for Control worms, Control worms + 10 μM TRO and Control worms + 20 μM TRO, respectively. All data were normalized to the untreated group; resulting fitness scores are shown and indicate that the molecule does not significantly increase the health of the control worms. NS: not significant by Student's *t*-test. Data represent mean ± s.e.m. (line thickness or error bar) with the N values listed above and are representative of three biological replicates that yielded similar results.

	no trodusquemine			with trodusquemine		
**Residue**	**^{1}H**	**^{15}N**	**Rel. Intensity (x 10^8)**	**^{1}H**	**^{15}N**	**Rel. Intensity (x 10^8)**
D1	-	-	-	-	-	-
A2	-	-	-	-	-	-
E3	8.24	120.41	3.16	8.24	120.40	1.99
F4	-	-	-		-	-
R5	7.93	123.71	1.57	7.93	123.71	0.92
H6	-	-	-	-	-	-
D7	8.11	121.82	2.15	8.11	121.82	1.33
S8	8.21	116.52	1.39	8.21	116.52	0.89
G9	8.32	110.64	1.49	8.32	110.62	0.88
Y10	7.73	119.98	1.56	7.73	119.97	0.93
E11	8.16	122.41	1.86	8.16	122.41	1.09
V12	7.86	121.09	1.45	7.86	121.08	1.01
H13	8.06	122.25	1.16	8.06	122.23	0.73
H14	-	-	-	-	-	-
Q15	8.20	121.37	1.73	8.20	121.39	1.09
K16	8.19	123.09	1.3	8.19	123.08	0.82
L17	8.06	123.90	1.61	8.06	123.90	1.05
V18	7.79	121.44	1.98	7.79	121.44	1.28
F19	8.06	124.37	1.77	8.06	124.36	1.13
F20	8.02	122.95	1.56	8.02	122.94	1.20
A21	8.03	126.11	1.72	8.04	126.12	1.13
E22	8.15	119.91	1.78	8.15	119.90	1.29
D23	8.22	121.76	1.74	8.21	121.77	1.01
V24	7.96	120.67	4.07	7.96	120.68	2.65
G25	8.34	111.65	1.57	8.34	111.64	0.77
S26	7.93	115.41	1.62	7.93	115.41	0.91
N27	-	-	-	-	-	-
K28	8.14	121.64	2.47	8.14	121.63	1.78
G29	8.21	109.44	1.6	8.20	109.45	0.89
A30	7.82	123.46	1.91	7.82	123.45	1.33
I31	7.96	120.67	4.07	7.96	120.68	2.65
I32	8.12	126.27	1.75	8.12	126.26	1.10
G33	8.28	113.04	1.58	8.28	113.02	1.27
L34	7.85	121.70	2.24	7.85	121.70	1.46
M35	8.22	121.97	2.41	8.22	121.95	1.20
V36	8.13	122.83	2.18	8.13	122.83	1.13
G37	8.54	114.21	1.79	8.54	114.22	0.85
G38	8.11	107.83	1.79	8.11	107.82	1.33
V39	7.71	119.73	2.33	7.71	119.73	1.32
V40	8.20	126.14	2.06	8.20	126.14	1.44
I41	8.25	127.65	2.43	8.25	127.65	1.84
A42	7.83	113.60	2.12	7.83	113.60	1.56

Supplementary Table 1. Raw HSQC data used for Supplementary Figure 3. Chemical shifts are in ppm, relative intensities were calculated using Sparky and are in arbitrary units. Normalized chemical shirt perturbations were computed using the relationship $\Delta\delta = \sqrt{(\Delta\delta_{HN})^2 + (\frac{\Delta\delta_N}{6.4})^2}$. [4]

Supplementary Note 1: Microscopic analysis of the effects of trodusquemine on Aβ$_{42}$ aggregation.

Analysis of the unseeded kinetic curves was carried out to probe the effects of trodusquemine on the product of rate constants involving primary pathways (k_+k_n) and secondary pathways (k_+k_2). This product can be further decoupled by analyzing the seeded kinetic curves where specific individual microscopic processes in the aggregation process can be negated, e.g. primary nucleation at low and high concentrations of seed fibrils, or become dominant, e.g. elongation at high seed concentrations. We found from this approach that the unseeded aggregation traces in the presence of different concentrations of trodusquemine could be closely described by varying the rate constants associated with secondary pathways (Fig. 1b). Indeed, the effects on k_+k_2 are in excellent agreement with the macroscopic kinetic data, as this product of rate constants was observed to be increased with a clear dose dependence, indicating an enhancement by a factor of approximately 5 in the presence of an equimolar ratio of Aβ$_{42}$-to-trodusquemine (Fig. 1f). Moreover, aggregation traces in the presence of 5% seed fibrils were well-described by fitting for k_2 (Fig. 1c). By contrast, fitting the unseeded and seeded kinetic traces by modifying globally just the rate of primary nucleation resulted in much lower quality fits in comparison to fitting globally for k_2 (Supplementary Figure 2). These results confirm the conclusion that the acceleration of aggregation observed in the presence of trodusquemine cannot be attributed to an increase in the rate of primary nucleation.

We then sought to distinguish between the relative contributions of secondary nucleation and elongation to the observed increase in k_+k_2. Experimental traces in the presence of a high concentration of seed fibrils reveal that trodusquemine causes only a small increase in the rate of elongation, as the half-time of the aggregation of Aβ$_{42}$ was reduced by a factor of 0.7 in the presence of an equimolar concentration of the molecule (Fig. 1d). Indeed, the aggregation traces for Aβ$_{42}$ in the presence of trodusquemine at ratios of 10:1 and 3:1 (Aβ$_{42}$-to-trodusquemine) were not significantly changed in the presence of 25% seed fibrils (Fig. 1d); however, in the presence of 5% seed fibrils, where secondary nucleation is rate-limiting, trodusquemine caused a discernable increase in the rate of aggregation (Fig. 1c). These results indicate that the alteration of the rate of elongation cannot by itself recapitulate the experimental data. As explained in the text, the lengths of the final fibrillar products measured by AFM are highly consistent with the

conclusion that trodusquemine increases the rate of monomer-dependent secondary nucleation (see main text, Fig. 2b).

Supplementary Note 2: Consideration of fibril height (AFM) and width (TEM).

A close inspection of the height distribution obtained with $A\beta_{42}$ in the absence of trodusquemine reveals a heterogeneous distribution with at least two populations of fibrillar aggregates having smaller (2-4 nm) and larger (4-7 nm) heights (Fig. 2f). We note that the difference in the average cross-sectional height and width of the fibrils measured with AFM and TEM, respectively, is in part a result of their non-cylindrical cross-sectional symmetry, which has an aspect ratio > 1, as observed when, for instance, an amyloid fibril adopts a twisted ribbon conformation[5]. Moreover, the addition of uranyl acetate increases slightly the measured width of each fibril[6], whereas the AFM technique causes a minor underestimation of fibril heights due to sample deformation[2,7]. Despite these differences, both methods demonstrate that the presence of trodusquemine during the process of $A\beta_{42}$ fibril formation results in the production of fibrils with increased cross-sectional dimensions.

Supplementary Note 3: TEM measurements at earlier stages of $A\beta_{42}$ aggregation.

To assess the effects of trodusquemine on earlier stages of Aβ42 fibril formation, in an additional set of experiments, TEM samples were prepared as previously described and deposited at t=1h, a time when $A\beta_{42}$ exits the lag phase of aggregation in the absence of trodusquemine and reaches its half-time of aggregation when co-incubated with an equimolar concentration of $A\beta_{42}$-to-trodusquemine. Fibrils formed in the presence of trodusquemine were characterized by the presence of branching aggregates, which suggests that the molecule potentiated nucleation at the fibril surface, a finding that was not observed for $A\beta_{42}$ after 1 h of aggregation in the absence of the molecule (Supplementary Figure 7). Indeed, such structures were also not observed in the plateau phase of the aggregation reaction for $A\beta_{42}$ in the absence of trodusquemine when measured by AFM and TEM (Fig. 2), indicating that the changes observed after 1 h of aggregation in its

presence are not related to differences in the fibril mass fraction. We note, in particular, that this pattern of fibril formation in the presence of trodusquemine at early stages in the aggregation reaction is not consistent with an increase in the rates associated with primary nucleation or elongation.

Supplementary Note 4: Determination of the composition and concentration of oligomers necessary to observe toxicity in human neuroblastoma cells.

We first sought to determine the conditions in which $A\beta_{42}$ oligomers are cytotoxic to human neuroblastoma cells in a reproducible manner. Oligomers were produced as previously described[3] using monomeric $A\beta_{42}$ as a recombinant or synthetic (Sigma Aldrich, MO, USA) form and subsequently added to cell culture media at a concentration of 12 μM (monomer equivalents). The viability of neuroblastoma SH-SY5Y cells was found to be reduced by 32.5±3.1% and 37.2±4.2% when incubated with the oligomers generated from synthetic or recombinant $A\beta_{42}$, respectively, as measured by the ability of the cells to reduce 3-(4,5-dimethylthiazol-2-yl)-2,5-diphenyltetrazolium bromide (MTT) (Supplementary Figure 8), indicating that the toxicity of the ADDLs is not significantly different when produced from these sources. Indeed, it has been shown that ADDLs generated from brain-derived and synthetic peptide have similar masses, isoelectric points, recognition by conformation-sensitive antibodies and binding to cultured primary neurons and toxicities[8].

Following this observation, we elected to generate the oligomers from synthetic $A\beta_{42}$. We then further tested the ability of the cells to reduce MTT in the presence of 1 μM $A\beta_{42}$ oligomers in order to minimize the concentrations of trodusquemine administered to the cells, as the molecule alone can cause a decrease in cellular viability (Fig. 3a). At over an order of magnitude lower concentration of oligomers, a significant toxic effect of the aggregates was observed as the cellular viability was found to be decreased by 23.2±2.4% (Fig. 3a).

In an additional set of measurements, we sought to characterize in greater detail the heterogeneity within our $A\beta_{42}$ oligomer preparation using dynamic light scattering. Oligomers were produced as previously described[3] and diluted to a concentration of 5 μM in 50 mM Tris buffer, 100 mM NaCl,

pH 7.4. An average oligomer diameter of approximately 22 nm and ranging from 13-50 nm was observed (Supplementary Figure 8), The heterogeneity of these species may explain why significantly higher concentrations of these aggregates are necessary to observe toxicity to cells in comparison to oligomers produced during the on-pathway aggregation reaction of $A\beta_{42}$.

Supplementary References

1. Roche, J., Shen, Y., Lee, J. H., Ying, J. & Bax, A. Monomeric Aβ1–40 and Aβ1–42 peptides in solution adopt very similar Ramachandran map distributions that closely resemble random coil. *Biochemistry* **55,** 762–775 (2016).
2. Ruggeri, F. S. *et al.* Nanoscale studies link amyloid maturity with polyglutamine diseases onset. *Sci. Rep.* **6,** 31155 (2016).
3. Lambert, M. P. *et al.* Vaccination with soluble Abeta oligomers generates toxicity-neutralizing antibodies. *J. Neurochem.* **79,** 595–605 (2001).
4. Mulder, F. A. A., Schipper, D., Bott, R. & Boelens, R. Altered flexibility in the substrate-binding site of related native and engineered high-alkaline Bacillus subtilisins. *J. Mol. Biol.* **292,** 111–123 (1999).
5. Adamcik, J. *et al.* Measurement of intrinsic properties of amyloid fibrils by the peak force QNM method. *Nanoscale* **4,** 4426–4429 (2012).
6. Gras, S. L., Waddington, L. J. & Goldie, K. N. Transmission Electron Microscopy of Amyloid Fibrils. In: Hill, A., Barnham, K., Bottomley, S. & Cappai, R. (eds) Protein Folding, Misfolding, and Disease. Methods in Molecular Biology (Methods and protocols), vol 752. Humana Press, Totowa, NJ (2011).
7. Ruggeri, F. S., Habchi, J., Cerreta, A. & Dietler, G. AFM-based single molecule techniques: unraveling the amyloid pathogenic species. *Curr. Pharm. Des.* **22,** 3950–3970 (2016).
8. Gong, Y. *et al.* Alzheimer's disease-affected brain: Presence of oligomeric Aβ ligands (ADDLs) suggests a molecular basis for reversible memory loss. *Proc. Natl. Acad. Sci. USA* **100,** 10417–10422 (2003).
