# Supplementary material for: Trodusquemine enhances Aβ42 aggregation but suppresses its toxicity by displacing oligomers from cell membranes
Source: Nat Commun. 2019 Jan 15;10:225. doi: 10.1038/s41467-018-07699-5 (PMC6333784; doi:10.1038/s41467-018-07699-5)
Supplement: Supplementary file 2 — Description of Additional Supplementary Files [file 41467_2018_7699_MOESM2_ESM.docx]

**Title:** Supplementary Movie 1.
**Description:** AD worms incubated from the L4 stage of development in the absence of trodusquemine. Video was recorded at day 5 of adulthood.

**Title:** Supplementary Movie 2.
**Description:** AD worms incubated from the L4 stage of development in the presence of a 20 µM dose of trodusquemine. Video was recorded at day 5 of adulthood.

**Title:** Supplementary Movie 3.
**Description:**Control worms incubated from the L4 stage of development in the absence of trodusquemine. Video was recorded at day 5 of adulthood.

**Title:** Supplementary Movie 4.
**Description:** Control worms incubated from the L4 stage of development in the presence of a 20 µM dose of trodusquemine. Video was recorded at day 5 of adulthood.
